# Assessment of hepatic transporter function in rats using dynamic gadoxetate-enhanced MRI: a reproducibility study

**DOI:** 10.1007/s10334-024-01192-5

**Published:** 2024-08-06

**Authors:** Ebony R. Gunwhy, Catherine D. G. Hines, Claudia Green, Iina Laitinen, Sirisha Tadimalla, Paul D. Hockings, Gunnar Schütz, J. Gerry Kenna, Steven Sourbron, John C. Waterton

**Affiliations:** 1 Division of Clinical Medicine, School of Medicine and Population Health, University of Sheffield, Polaris, 18 Claremont Crescent, Sheffield, S10 2TA UK; 2 GSK, Collegeville, Collegeville, P.A. USA; 3 MR & CT Contrast Media Research, Bayer AG, Berlin, Germany; 4 Antaros Medical, GoCo House, Mölndal, Sweden; 5 Sanofi-Aventis GmbH, Frankfurt, Germany; 6 Institute of Medical Physics, University of Sydney, Sydney, Australia; 7 Chalmers University of Technology, Gothenburg, Sweden; 8 Bioxydyn Ltd, St. James Tower, Manchester, UK; 9 Centre for Imaging Sciences, Division of Informatics Imaging & Data Sciences, School of Health Sciences, Faculty of Biology Medicine & Health, University of Manchester, Manchester, UK

**Keywords:** Chemical and drug induced liver injury, Rats, Reproducibility of results, Gadolinium ethoxybenzyl DTPA, Magnetic resonance imaging

## Abstract

**Objective:**

Previous studies have revealed a substantial between-centre variability in DCE-MRI biomarkers of hepatocellular function in rats. This study aims to identify the main sources of variability by comparing data measured at different centres and field strengths, at different days in the same subjects, and over the course of several months in the same centre.

**Materials and methods:**

13 substudies were conducted across three facilities on two 4.7 T and two 7 T scanners using a 3D spoiled gradient echo acquisition. All substudies included 3–6 male Wistar-Han rats each, either scanned once with vehicle (*n* = 76) or twice with either vehicle (*n* = 19) or 10 mg/kg of rifampicin (*n* = 13) at follow-up. Absolute values, between-centre reproducibility, within-subject repeatability, detection limits, and effect sizes were derived for hepatocellular uptake rate (*K*^trans^) and biliary excretion rate (*k*_bh_). Sources of variability were identified using analysis of variance and stratification by centre, field strength, and time period.

**Results:**

Data showed significant differences between substudies of 31% for *K*^trans^ (*p* = 0.013) and 43% for *k*_bh_ (*p* < 0.001). Within-subject differences were substantially smaller for *k*_bh_ (8%) but less so for *K*^trans^ (25%). Rifampicin-induced inhibition was safely above the detection limits, with an effect size of 75 ± 3% in *K*^trans^ and 67 ± 8% in *k*_bh_. Most of the variability in individual data was accounted for by between-subject (*K*^trans^ = 23.5%; *k*_bh_ = 42.5%) and between-centre (*K*^trans^ = 44.9%; *k*_bh_ = 50.9%) variability, substantially more than the between-day variation (*K*^trans^ = 0.1%; *k*_bh_ = 5.6%). Significant differences in *k*_bh_ were found between field strengths at the same centre, between centres at the same field strength, and between repeat experiments over 2 months apart in the same centre.

**Discussion:**

Between-centre bias caused by factors such as hardware differences, subject preparations, and operator dependence is the main source of variability in DCE-MRI of liver function in rats, closely followed by biological between-subject differences. Future method development should focus on reducing these sources of error to minimise the sample sizes needed to detect more subtle levels of inhibition.

**Supplementary Information:**

The online version contains supplementary material available at 10.1007/s10334-024-01192-5.

## Introduction

Drug-induced liver injury (DILI) is a serious public health problem and a major concern in drug development. The WITHDRAWN database lists 60 drugs withdrawn or discontinued for DILI, which was fatal for at least 26 of the listed 60 [1]. Impaired liver function also plays a key role in drug-drug interactions (DDI), which remain a cause for concern in drug development and are often caused by hepatic transporter inhibition or induction. A critical challenge in the management of DILI and DDI is the need for better diagnostics to identify the risk earlier in the drug development lifecycle and optimise patient selection for treatment.

Dynamic contrast-enhanced MRI (DCE MRI) with gadoxetate provides potential biomarkers of DILI or DDI occurring through hepatobiliary transporter inhibition [2]. Gadoxetate is a liver-specific contrast agent that is actively taken up into hepatocytes with biliary clearance of 50% in humans [3] and 70% in rats [4]. Gadoxetate is a known substrate for multiple influx and efflux transporters which are responsible for the liver specificity and biliary clearance of this agent [5–10]. Since gadoxetate and “perpetrator” drugs are both substrates of these transporters, an opportunity exists for using gadoxetate DCE-MRI to measure liver transporter inhibition or alteration, and thereby predict the risk of DILI or DDI [2, 11, 12].

The use of gadoxetate DCE-MRI as a biomarker for DILI or DDI risk is supported by an increasing body of evidence both in disease models and in humans [9, 13–20]. In a landmark paper published in 2018, Karageorgis et al. [21] reported on the first multi-centre study using gadoxetate DCE-MRI to measure liver transporter function using the method of Ulloa et al. [2]. The study used a case–control design with different animals given either a vehicle or a potent inhibitor before gadoxetate DCE-MRI. The study showed compelling evidence that inhibition of liver uptake and excretion can be detected with confidence. The data also showed between-centre biases in absolute values of uptake and excretion rates, though the relative differences between treatment and control groups were more consistent between centres.

Future development of the assay can potentially reduce the between-centre biases further, and thereby reduce the sample sizes needed to detect a given level of drug-induced inhibition of liver function. To inform such further development, this study aimed to unravel the sources of variability and identify the main sources of the observed between-centre bias, both in the absence of drugs and in the presence of a known strong inhibitor. Rifampicin was selected due to its potential for causing DILI, as determined from previous in vitro [22] and in vivo analysis [21]. Data were collected across three centres, at two field strengths, and over time periods separated by 2–19 months. In a subset of data, repeated measurements were performed in each subject.

## Materials and methods

### Assay development

The assay used in this study was developed by the TRISTAN project -an international collaborative private-public partnership aiming, among others, to develop standardised assays for hepatic transporter assessments in rats and humans. The standardised assay in rats was developed in consensus after a comparison of methods in literature and preliminary acquisitions and sensitivity analyses, with the results published and presented at relevant society meetings [23–30]. Subsequently, a systematic program of technical and biological validation studies was initiated to characterise the assay in terms of its parameter uncertainty, sensitivity to effects of drugs with different potency, and ability to detect adaptations after repeat dosing. Differences with the assay used in Karageorgis et al. [21] include the use of a model-based input function [30] and allowing for different relaxivity values between extra- and intracellular spaces. The assay is specified in the supplementary material which includes sufficient detail for independent replication.

### Study design

Data were collected prospectively from eight substudies, with side-line data included retrospectively from five additional substudies (see Table 1 for more details). This resulted in 13 substudies available for analysis, covering:

1. Three centres: D, E, and G (see Table 2 for hardware specifications per centre)
2. Two MRI field strengths: 4.7 T, 7 T
3. Eight-time periods between experiments (substudies): 2 months, 10 months, 12 months, 13 months, 14 months, 15 months, 16 months, 19 months
4. Two treatments: saline and rifampicin

**Table 1:** Summary of subjects included, sorted by centre, field strength, design, and treatment

Substudy	Centre	Field strength (T)	N_subjects_ / group	Baseline (Day 1)	Follow-up (Day 2)	Study date	Study type
1	D	4.7	3	Saline	Saline	July 2018	P
2	E	7	3	Saline	Saline	Sept. 2018	P
3	G	7	3	Saline	Saline	July 2018	P
4	G	4.7	4	Saline	Saline	Sept. 2019	P
1	D	4.7	3	Saline	Rifampicin	July 2018	P
2	E	7	3	Saline	Rifampicin	Sept. 2018	P
3	G	7	3	Saline	Rifampicin	July 2018	P
4	G	4.7	4	Saline	Rifampicin	Sept. 2019	P
5	E	7	6	Vehicle	–	Sept. 2019	R
6	E	7	6	Vehicle	–	Oct. 2019	R
7	D	4.7	6	Vehicle	–	Sept. 2019	R
8	G	4.7	6	Vehicle	–	Nov. 2019	R
9	G	7	4	Vehicle	–	Oct. 2019	R
10	G	7	6	Vehicle	–	May 2019	R
11	G	7	6	Vehicle	–	Nov. 2019	R
12	G	4.7	4	Saline	–	Sept. 2019	R
13	G	7	3^a^	Saline	–	Sept. 2019	P
13	G	7	3^b^	Saline	–	Sept. 2019	P
13	G	4.7	3^a^	–	Saline	Sept. 2019	P
13	G	4.7	3^b^	–	Saline	Sept. 2019	P

Follow-up treatments on Day 2 for substudies 5–12 are beyond the scope of this paper. Substudies 1–4 have been duplicated to differentiate between those treated with saline at follow-up and those with rifampicin

^a^These subjects were imaged by the same centre using 7T at baseline and 4.7T at follow-up

^b^These subjects were imaged by the same centre using 4.7T at baseline and 7T at follow-up

P = prospective; R = retrospective

**Table 2:** MRI specifications per centre

Centre	Spectrometer	Gradient strength / $mT\bullet {m}^{-1}$ (model)	Radiofrequency transmitter / receiver volume coil (i.d./mm)	Software
D	Biospec 47/20 USR Avance IIIHD	660 (B-GA12S HP)	Quadrature 200 MHz (72)	ParaVision 6.0.1
E	Biospec 70/30 USR Avance II	440 (B-GA12S)	Single channel 300 MHz (72)	ParaVision 6.0.1
G (4.7 T)	Biospec 47/40 Avance III	200 (B-GA12S)	Quadrature 200 MHz (90)	ParaVision 6.0.1
G (7 T)	Biospec 70/30 USR Avance III	300 (B-GA12)	Quadrature 300 MHz (90)	ParaVision 6.0.1

Each of the 13 substudies included between three to eight subjects, which were either scanned once (Day 1) or twice for repeatability assessment (Day 1, Day 2). Day 1 and Day 2 repeated MRI measurements were performed two to seven days apart, to ensure adequate wash-out of contrast agent and animal recovery of the anaesthesia between scans. During the baseline scan (Day 1) of each study, all subjects were administered saline/vehicle, while either saline/vehicle or another drug of interest was administered to subjects during the follow-up scan (Day 2). The date that each substudy was conducted is listed in Table 1. For each centre, time periods refer to the interval of time between the date that each separate substudy was conducted.

Substudies 1–4 comprise a multicentre test–retest study, where saline only was administered to half of the subjects at follow-up while rifampicin was administered to the other half. Substudies 5–12 comprise baseline saline/vehicle data from pharmacologic studies using the same protocol [25, 31]. Substudy 13 was a single-centre test–retest cross-over study, where six subjects were observed following the administration of saline in a scanner at 4.7 T on one day and then in a scanner at 7 T on an alternate day. This cross-over study was conducted to determine the effect of field strength on biomarker detection within the same subjects and at the same centre.

### Data acquisition

Data were acquired as described in more detail in the assay specification (Supplementary material, Sect.  1). Rats were purchased locally from Charles River Laboratories with a weight of 250 g at the time of ordering. Animals were provided enrichment, standard rat chow and water ad libitum, and were pair-housed with 12 h light/dark cycles. Following local veterinary advice, the air mixture was comprised of medical grade air, or combinations of 100% oxygen with either nitrogen or nitrous oxide. Towards the initiation of the exam, animals received saline or rifampicin at -60 min for the administration of gadoxetate, following the protocol reported in Karageorgis et al. [21].

**Fig. 1:**
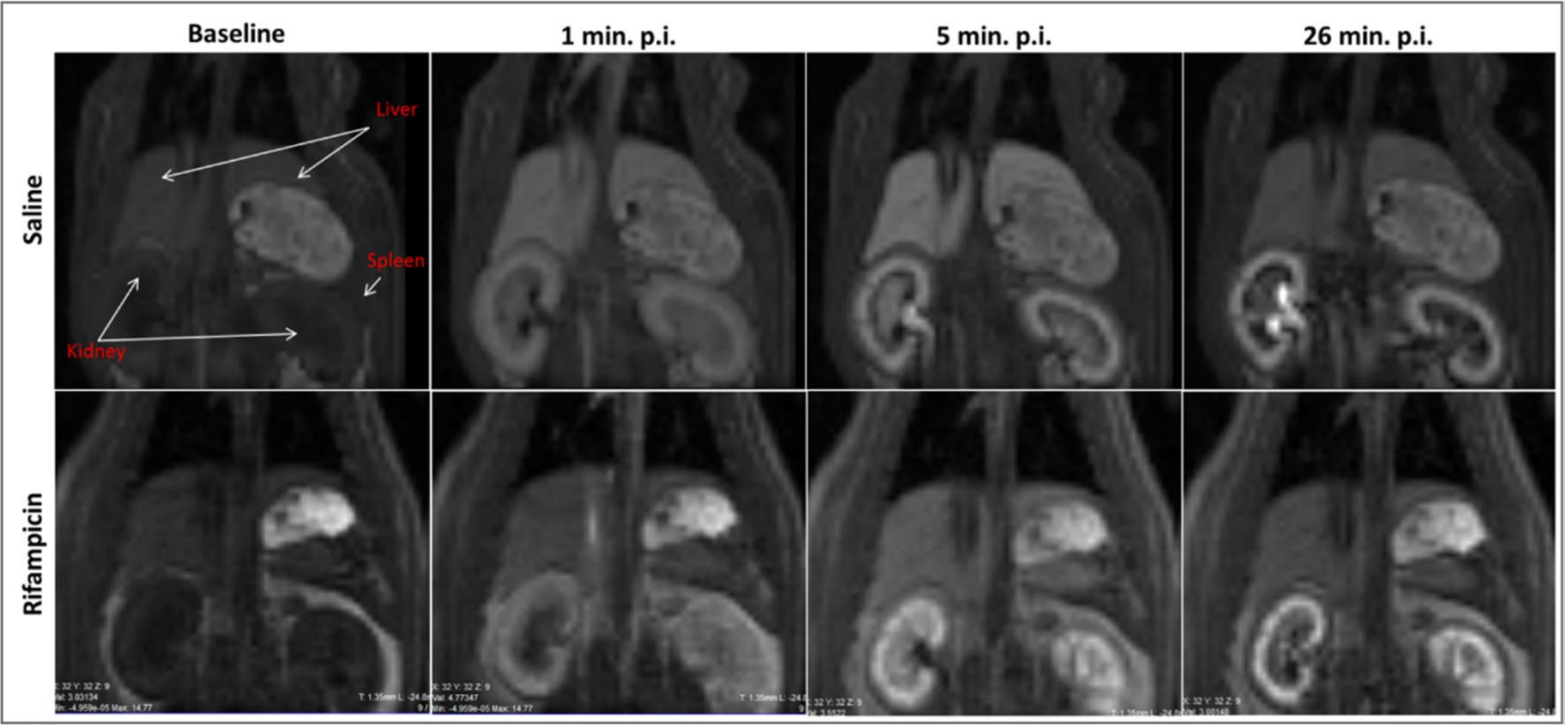
Sample MRI data comparing scans at multiple time points for a single rat after administration of saline (TOP) and rifampicin (BOTTOM)

MRI was performed using 4.7 T or 7 T Bruker (Ettlingen, Germany) scanners as shown in Table 1. DCE-MRI was acquired with a 3D Fast Low Angle Shot RF-spoiled gradient echo sequence (FLASH) with an oversampling of 6 and retrospective triggering (IntraGate, Bruker, Ettlingen, Germany) from a respiratory navigator measured in front of each FLASH pulse (slice thickness = 30 mm; FA = 2°). Imaging parameters included the following: TE/TR = 1.1/5.8 ms, 26 slices, slice thickness = 1.35 mm, FOV = 60 × 60 × 35 mm and a 64 × 64 × 26 matrix for a resolution of 0.94 × 0.94 × 1.35 mm, coronal orientation, readout in rostral-caudal direction, 6-fold phase oversampling and a flip angle of 30 degrees (centre E) or 20 degrees (centres D and G). For DCE, heparinised saline was placed in the tail vein catheter prior to the bolus of gadoxetate (see Supplementary material, Sect. 1.2). Gadoxetate Primovist or Eovist (Bayer AG, Berlin, Germany) was diluted 1:5 in saline and administered at 0.5 mL/kg (25 μmol/kg bodyweight). A separate phantom study [27] was performed prior to this study to characterise the effect of gadoxetate on T1. Relaxivity values were obtained from the literature [26] as described in the Supplementary material of this manuscript (Sect. 1.4). Any additional MRI parameters are also detailed in Sect. 1.4 of the Supplementary material. An example of DCE-MRI data acquired in this study is shown in Fig. 1.

### Data analysis

Region of interest (ROI) analysis was performed at the centre of acquisition for each rat using a version of the software PMI [32] tailored to the TRISTAN rat assay including detailed standard operating procedures (SOPs) and training of users by a central centre [26, 32]. Signal-time curves were exported from PMI to csv files and further processed by a Python script [33]. All signal-time curves have also been made available [34]. Rates for hepatic plasma clearance, *K*^trans^ [mL/min/mL] and biliary efflux, *k*_bh_ [mL/min/mL] of gadoxetate (i.e., the TRISTAN rat assay biomarker rate constants of interest) were then derived from the signal-time curves via tracer kinetic two-compartment modelling (see Supplementary material, Sect.  1.4).

Each substudy returned a single mean value for each of the two biomarkers *K*^trans^ and *k*_bh_, along with a 95% Confidence Interval (CI, defined as 1.96 × standard error).

Reference values for *K*^trans^ and *k*_bh_ were derived as the mean baseline value overall substudies, along with their CI. Reproducibility (of a single measurement) was quantified as the 95% tolerance interval (TI, defined as 1.96 × standard deviation) over substudies and expressed as a percentage of the mean. Repeatability (of a single measurement) was quantified for each of the substudies 1–4 (Table 1, rows 1–4) as the TI of baseline and follow-up saline data, expressed as a percentage of the mean. Rifampicin effect size (of a single measurement) was calculated for each of the substudies 1–4 (Table 1, rows 5–8) as the difference between saline value and inhibited value, expressed as a percentage of the saline value. Final values for repeatability and rifampicin effect size were determined as the mean (with CI) over the 4 substudies.

The absolute detection limit (%) for inhibition of any given biomarker was defined as the smallest change producing a TI that does not overlap with the CI of the saline benchmark value. The TI was estimated by applying the reproducibility error in saline. The relative detection limit (%) for inhibition of any given biomarker was defined as the smallest change producing a CI on the between-day difference that does not contain the value 0. The CI on the difference was estimated by applying the repeatability in saline, multiplied with $\sqrt{2}$ to account for the error in both terms. This quantity is also known as the repeatability coefficient (RC), a recommended metric for repeatability [35].

An analysis of sources of variability was performed on the data from substudies 1–4 using pairwise t-tests and analysis of variance. A value of *p* < 0.05 was considered significant for all statistical tests. As these analyses are intended to be exploratory, no adjustment was made for multiple comparisons. The *mixed_anova* function from the Python package *Pingouin* [36] was used to partition the sources of variability into between-substudy (reproducibility), between-day (repeatability), and between-subject components for a two-way mixed-effects model with a 2 × 3 study design. A one-way ANOVA was conducted to identify systematic differences between substudies. To help understand the impact of different variables in isolation, the Day 1 data were stratified by centre, field strengths, and time periods and tested for significant differences using a one-way ANOVA.

## Results

### Exclusions

All subjects survived until the end of the procedures without adverse effects. Eight subjects were excluded from analysis due to artefacts in Day 1 data, reducing the N_subjects_ to three subjects in substudy 10, three in substudy 13, and one subject in the repeatability data of substudy 2 (line 2 of Table 1). Since substudy 2 was left with only one set of valid repeatability data, it was excluded from single-substudy repeatability and rifampicin assessment. One subject was also excluded due to artefacts in Day 2 data, reducing the N_subjects_ with valid repeatability data to two in substudy 3 (line 3 of Table 1).

### Main results

**Fig. 2:**
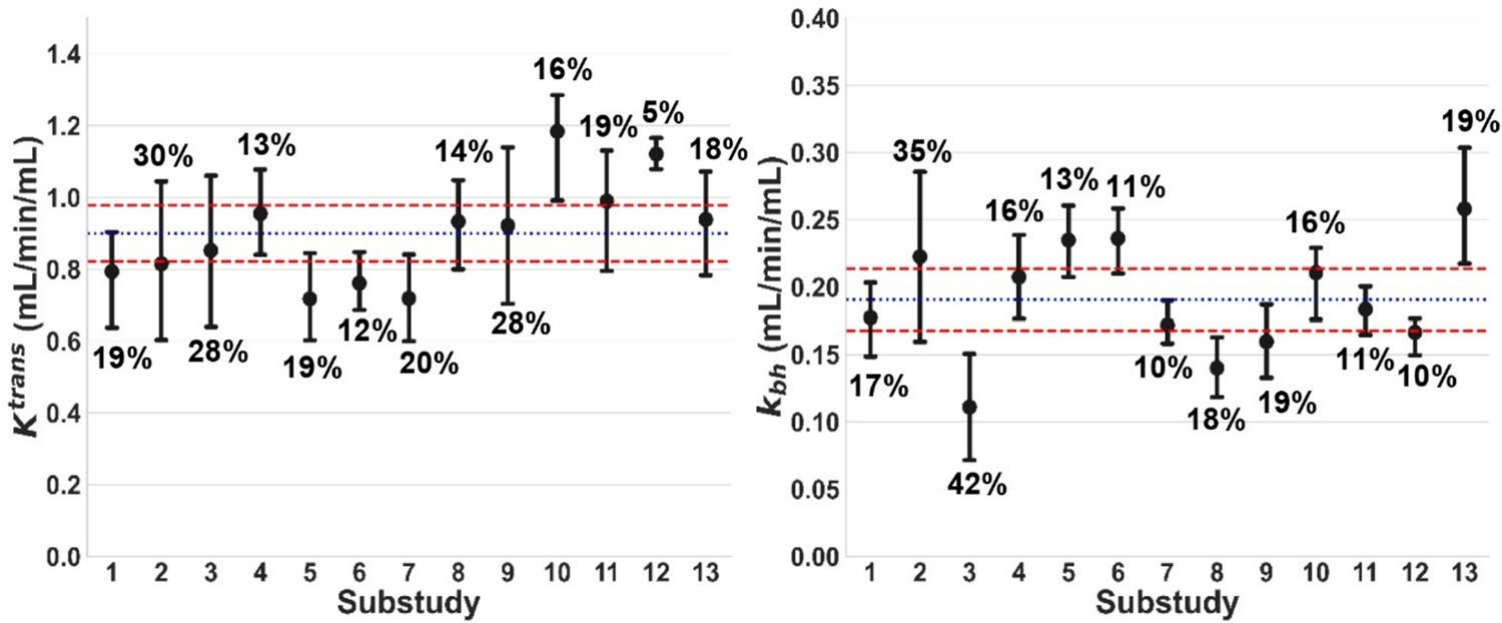
Distribution of baseline *K*^trans^ and *k*_bh_ [mL/min/mL] for all substudies. Error bars show mean values for each substudy and the 95% CI on the mean. The relative error on the mean is shown as a percentage above/below each error bar. Blue lines show benchmark values for each biomarker derived as the mean across all substudies. Red lines indicate the 95% CI on the mean

**Fig. 3:**
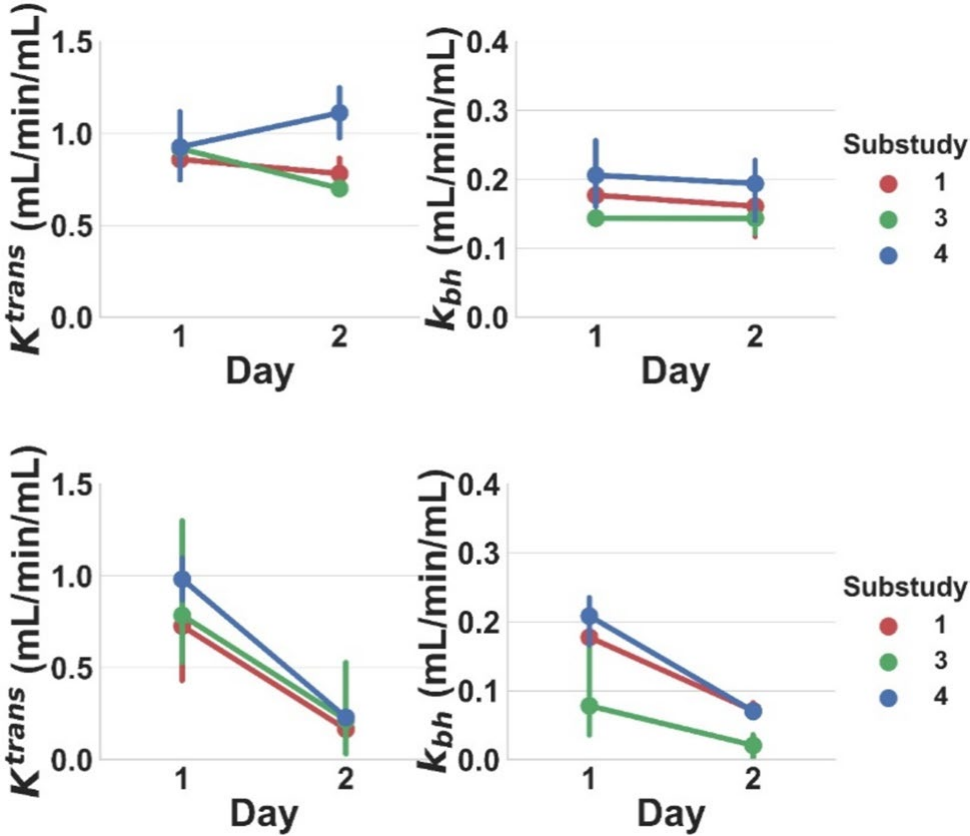
Baseline and follow-up *K*^trans^ and *k*_bh_ [mL/min/mL] values for the control group treated with saline on both days (top row), and the treatment group treated with saline at Day 1 and rifampicin at Day 2 (bottom row). Results are shown for all 3 included substudies. Lines connect the mean value at Day 1 and Day 2 for each substudy. Error bars indicate the 95% confidence interval on the mean

Figure 2 summarises the baseline values for all 13 substudies, showing significant differences between substudies for *K*^trans^ (*p* = 0.013) and *k*_bh_ (*p* < 0.001). Average values over all substudies were *K*^trans^ = 0.90 ± 0.08 mL/min/mL and *k*_bh_ = 0.19 ± 0.02 mL/min/mL. Two substudies produced a *K*^trans^ measurement outside of this reference range and three other substudies produced a *k*_bh_ measurement outside this reference range. Figure 3 shows Day 1 vs. Day 2 results for each substudy, showing clear systematic inhibition of *K*^trans^ and *k*_bh_ after rifampicin. Day 1 and Day 2 results under the same conditions are similar.

Reproducibility was 31% for *K*^trans^ and 43% for *k*_bh_; repeatability was 25% for *K*^trans^ and 8% for *k*_bh_. Inhibition must be above the absolute detection limit of 30% for *K*^trans^ and 37% for *k*_bh_ to be detectable from a single data point. The relative detection limit is substantially lower for *k*_bh_ (11%), but not for *K*^trans^ (35%). Rifampicin-induced inhibition was safely above this detection limit, with an effect size of 75 ± 3% in *K*^trans^ and 67 ± 8% in *k*_bh_. In absolute terms, rifampicin reduced *K*^trans^ significantly from 0.83 ± 0.15 to 0.20 ± 0.04 mL/min/mL and *k*_bh_ from 0.15 ± 0.08 to 0.05 ± 0.03 mL/min/mL.

### Sources of variability

Most of the variability in individual data was accounted for by biological between-subject (*K*^trans^ = 23.5%; *k*_bh_ = 42.5%) and between-centre (*K*^trans^ = 44.9%; *k*_bh_ = 50.9%) variability, substantially more than the between-day variation (*K*^trans^ = 0.1%; *k*_bh_ = 5.6%). All other sources of variability combined contributed 31.5% of the total variability in *K*^trans^ and 1.0% in *k*_bh_.

**Fig. 4:**
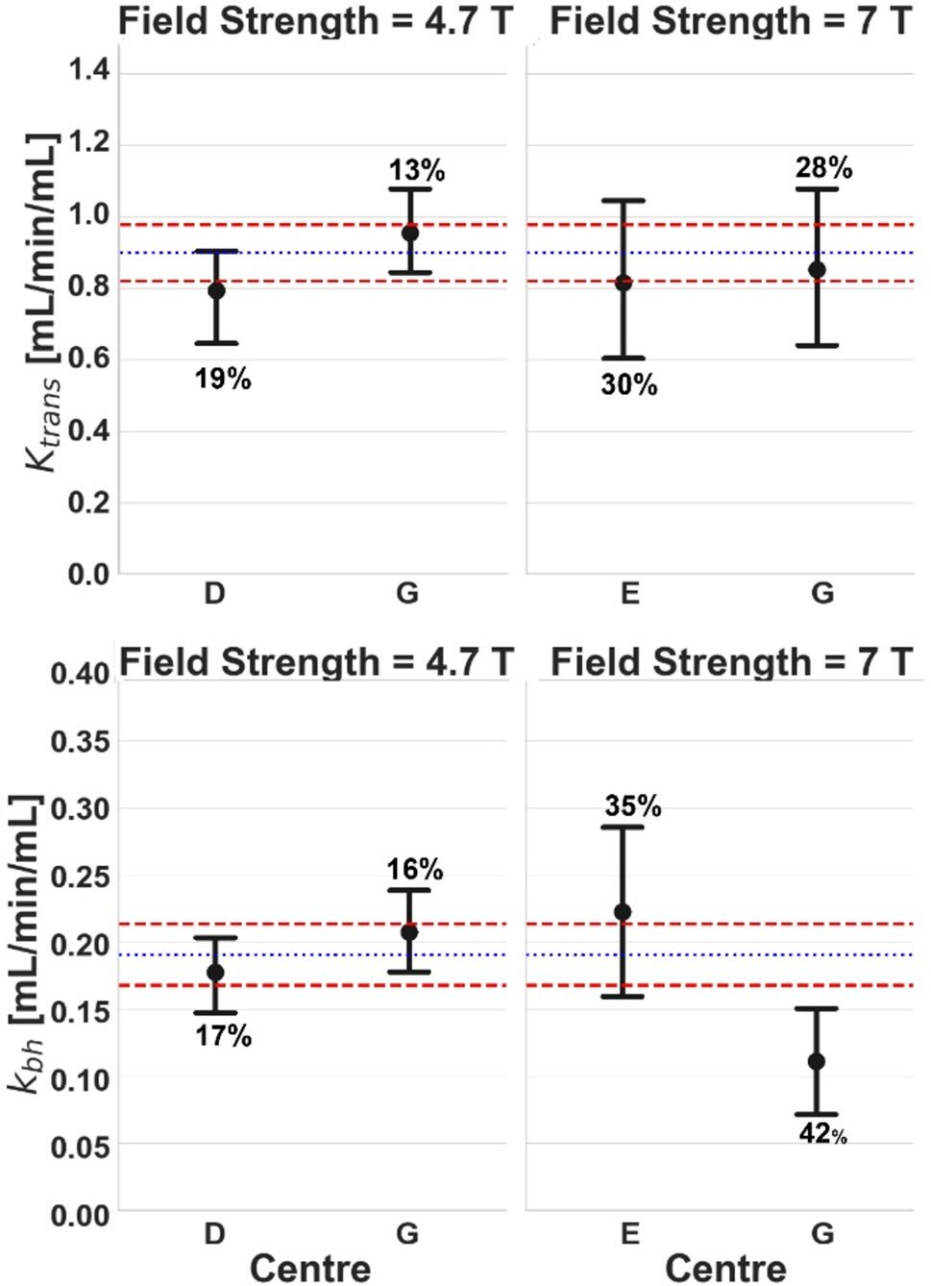
Comparison of baseline *K*^trans^ (top row) and *k*_bh_ (bottom row) [mL/min/mL] for different centres but at the same field strength: 4.7 T (left column) and 7 T (right column). Error bars and colour coding are the same as Fig. 2

**Fig. 5:**
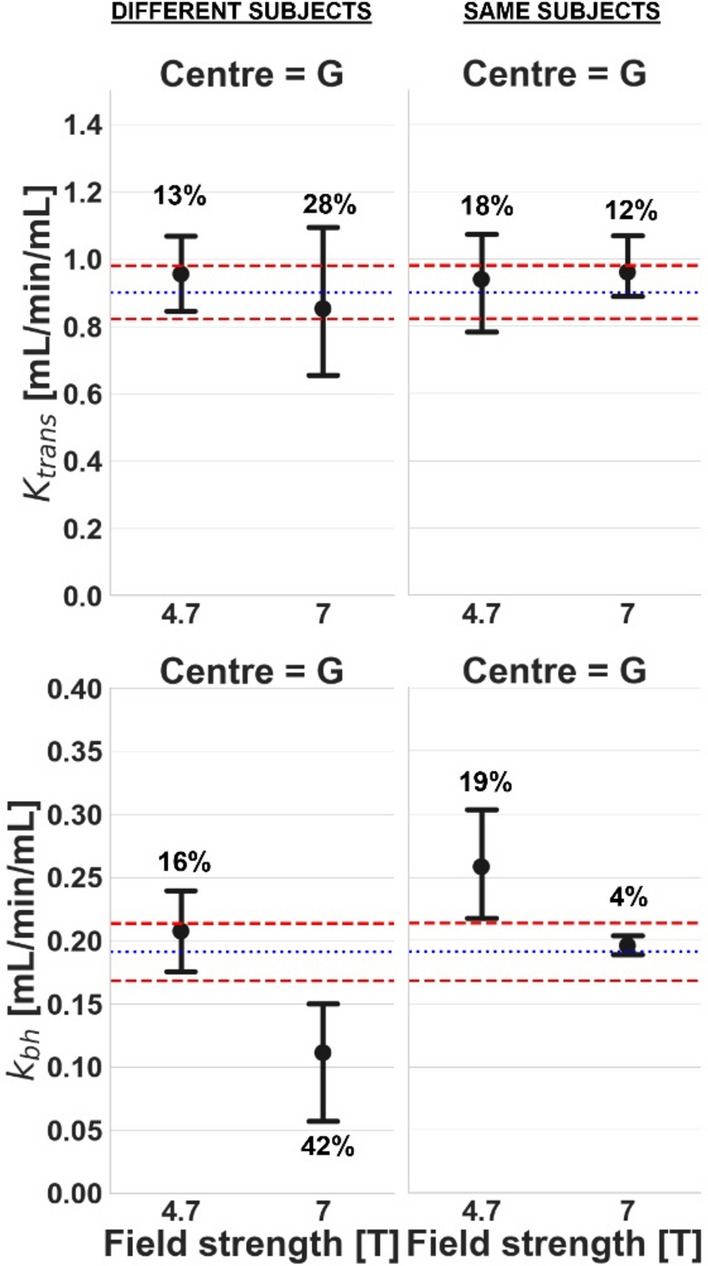
Comparison of baseline *K*^trans^ (top row) and *k*_bh_ (bottom row) [mL/min/mL] for different field strengths at the same centre. Results are shown for a study with different subjects (left column) and a study with the same subjects (right column). Error bars and colour coding are the same as Fig. 2

**Fig. 6:**
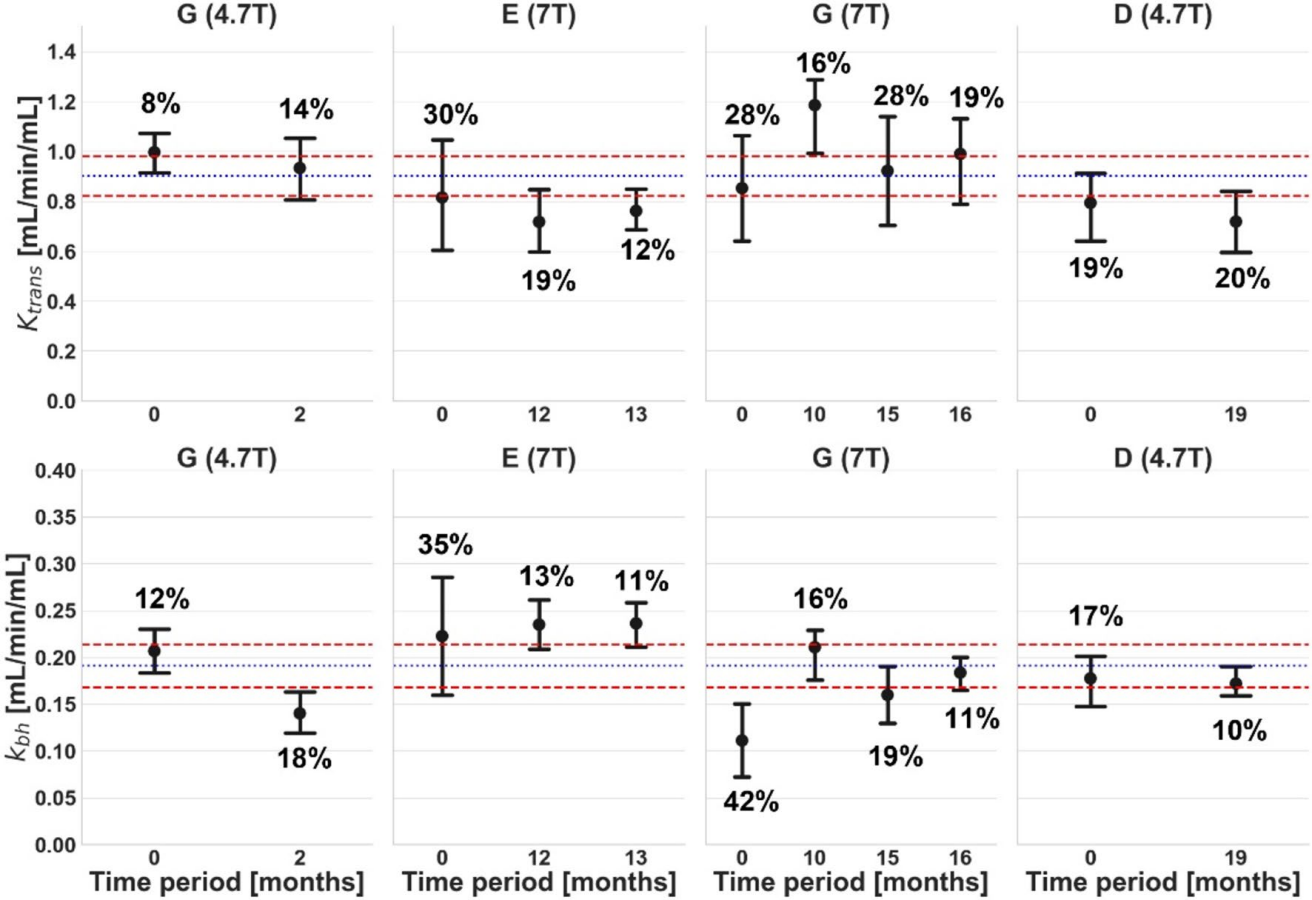
Comparison of baseline *K*^trans^ (top row) and *k*_bh_ (bottom row) [mL/min/mL] at the same scanner but at different time periods. Results are shown for centre G at 4.7 T (left), centre E at 7 T (middle left), centre G at 7 T (middle right), and centre D at 4.7 T (right). Error bars and colour coding are the same as Fig. 2

Figures 4–6 show the effect of individual variables (centre, field strength, and time point) after correcting for the others.

No significant differences were found for *K*^trans^. Centres E and G, both operating at 7 T in the same time period, produced significantly different values for *k*_bh_ (Fig. 4).

In centre G, which operated at both 7 T and 4.7 T, significant differences in *k*_bh_ were found between field strengths (Fig. 5), even if measurements were performed in the same subjects. When the experiments were repeated several months apart at the same centre and field strength (Fig. 6), significant differences were observed in *k*_bh_ after 2 months (centre G, 4.7 T) and after 10 months (centre G, 7 T).

## Discussion

Reproducibility is a critical challenge for DCE-MRI, which currently hampers its use in biomarker-driven drug discovery or identification of safety risks (“de-risking”). The purpose of the current paper was to inform future method development by unravelling the sources of between-centre variability. For this purpose, 89 rat datasets were analysed from 13 separate studies at three institutions with four MRI scanners. Detailed application notes are provided in supplementary material to allow independent implementation and replication of the results.

The data demonstrated that the smallest detectable inhibition of absolute rate constants is about 2 times lower than the effect size of a potent inhibitor. The smallest detectable change in the relative rate constants values is similar for uptake but 6 times less for excretion. This suggests that the assay is sufficiently robust to detect the effect of potent inhibitors and should also be sufficient for substantially weaker inhibitors. Studies with different test compounds of various levels of potency are underway to determine exactly which level of inhibition is detectable. To avoid overinterpretation of results, the reproducibility reported in this study needs to be accounted for when comparing absolute values to the reference range derived in this study.

The detection limit on relative values for *k*_bh_ is about 6 times lower than that on absolute values. This shows that, in its current form, the assay is best applied by comparing a baseline assessment (saline) against an assessment with the test drug in the same population, performed on consecutive days, and reporting the relative changed caused by the drug rather than the absolute values in the presence of the test compound. The trade-off is the need for an extra control experiment in the same subjects, which at the preclinical level is a relatively small additional burden considering the gain in power of the assay.

Reference values produced in this study differ by several orders of magnitude from values reported by Karageorgis et al. [21]. However, theoretical analysis [37] has demonstrated that the latter are overestimated by a factor of 1000 due to a mistake in units, and are defined relative to a different concentration than uptake rates presented in this paper. After correcting for those differences, the values reported in Karageorgis et al. [21] are *K*^trans^ = 1.0 mL/min/mL and *k*_bh_ = 0.07 mL/min/mL. The *K*^trans^ value reported in [21] is very close to our reference range of 0.90 ± 0.08 mL/min/mL, however, *k*_bh_ is considerably outside of the reference range of 0.19 ± 0.02 mL/min/mL. Ignoring differences in definition, relative changes under rifampicin reported in Karageorgis et al. are broadly similar for *K*^trans^ (70% vs. 75%) but are a factor of 1.8 lower for *k*_bh_ (38% vs. 67%). A reanalysis of the data from Karageorgis et al. [21] using the modelling approach used in our own work would be required to determine the exact reason for these differences. However, this was beyond the current scope of the work presented in this study. Potentially this higher sensitivity to change can be traced back to a series of refinements introduced in the assay, such as the use of accurate measurements of gadoxetate relaxivities in blood and plasma [26], modelling that accounts for relaxivity differences between liver and blood, or the use of a model-based input function. A more detailed analysis of sources of variability and sensitivity of the results to these changes would be needed to unravel the different contributions of these changes and to understand whether they can explain the differences observed.

Variability analysis indicates that a reduction of biological variability due to environmental conditions should be a priority for future improvement of the assay. The analysis of variances shows that the between-subject variability in any single centre and between-centre variability are large contributing factors to the variability of the assay. This indicates that experience with biological factors from cancer models does not translate to DILI and DDI models. Indeed, Ng et al. measured *K*^trans^ three times in rat tumours [38] and found inter-rat variance was 28% of the total variability, while intra-rat variance comprised 72%, indicating that the repeatability error was much larger than the heterogeneity of the population.

The aim of our assay was to compare average biomarker values per centre rather than individual values per subject. However, between-subject analyses were included to help understand possible sources of error. In the individual comparisons, the between-subject error was the second-largest contributor to variability (*K*^trans^ = 23.5%; *k*_bh_ = 42.5%). It is therefore plausible that biological variability between populations also contributes to the between-centre variability. In terms of the technical sources of variability, the analysis indicates that two of the contributing factors investigated (centre, field strength) can have a substantial effect. This suggests that future efforts on reducing the overall technical uncertainties of the assay need to focus on all three variables. Another issue is the relatively high number of exclusions due to artefacts or fit failures. While this can be compensated in animal studies by increasing the number of subjects, this indicates an inherent instability of the assay that should be reduced in future improvements.

The between-centre variance can potentially be reduced by standardised central training of operators, more detailed standard operating procedures, and removal of variables such as choice of anaesthesia, number of subjects and subjective criteria for exclusions. In terms of field strength, while known differences such as baseline *R*_1_ and relaxivity are corrected for, and the impact on *K*^trans^ appears small, there remains a significant impact on *k*_bh_. Comparison to the benchmark values suggests that the 7 T results for *k*_bh_ rather than the 4.7 T results are an outlier, potentially indicating typical high-field effects such as increased susceptibility artefacts are responsible. Closer investigation of artefacts at different field strengths is needed to identify the source of these errors and identify practical solutions. In terms of changes over time, the data did not clearly indicate a trend or drift in values over any of the time scales. However, there was significant variability in *k*_bh_ values measured over time periods of 0–2 months and 0–16 months. The causes are not clear at this moment but–apart from biological factors - could potentially be explained by staff turnover or hardware/software upgrades. This indicates that these factors need to be carefully recorded and controlled in future experiments.

A barrier to the further development and deployment of gadoxetate DCE assays for liver transporter function is the wide range of methods and approaches for acquisition and analysis. This leads to sometimes orders of magnitude difference between published values, and a difficulty in identifying clear relationships between data from different studies [37]. Independent replication of the assays is effectively prohibited due to the lack of detailed standard operating procedures for acquisition and analysis, hardware specifications, sequence cards, software for analysis or implementations of modelling sections. This highlights a clear need for a well-defined, standardised, and authoritative assay that can be replicated independently, is broadly acceptable by the community and produces well-defined biomarkers [39, 40] characterised by benchmark values and their uncertainties. The assay introduced in this study will be versioned methodically to establish clear benchmarks but also account for any future improvements in acquisition or analysis methodology. The assay also served as a critical step in a translational pathway and led to the development of a compatible human assay. First validation studies of the human assay are underway and on 25^th^ February 2021, a letter of intent on the human assay was accepted into the formal biomarker qualification program of the Food and Drug Administration (FDA) [41].

## Limitations

Repeatability measurements should ideally be performed under identical conditions, which requires a ‘coffee-break’ type experiment with a short interval between both scans. This is not feasible in this context because sufficient time must be allowed for gadoxetate to fully clear from the body. Therefore, assessments are performed on successive days, which may cause some added variability due to recovery from anaesthesia or other physiological impacts of the previous day’s measurement. Furthermore, the time between successive scans varied for each centre (ranging from two to seven days) for practical reasons due to MRI scanner availability. This may have introduced more variability in the between-subject variance observed in this study.

Reproducibility assessments are ideally performed in the same subjects so that the effect of experimental variables can be separated from population heterogeneity. In rat studies this is not feasible as the transport, changes in housing and diet, and time required for transfer between centres is likely to cause significant changes. Hence assessments at different centres were performed on different subjects, which means that the effects of population heterogeneity could not be fully distinguished.

The confidence intervals on the absolute values for *K*^trans^ and *k*_bh_ show that the current sample size is sufficient to estimate these with a high degree of confidence. Since the analysis reveals systematic differences between centres, field strengths, and time periods, increasing the sample size further will not fundamentally affect the conclusions on sources of variability. This confirms that the study was adequately powered for the primary objective.

However, the sample size of this study is insufficient to provide a high degree of confidence on the parameter errors, such as the reproducibility or repeatability errors provided in this paper, or the detection limits on absolute and relative values. Reliable estimates of the “error on the error” would typically require substantially larger studies. For this reason, confidence intervals on the errors themselves are not provided in this paper, and their values should be seen as order-of-magnitude estimates rather than reliable benchmarks for data interpretation in future studies.

Not all data presented in this paper are prospective, as some studies were planned after the first results were analysed to fill in gaps in the data. Also, while acquisitions at different centres were performed independently by different teams, the nature of the project involved frequent discussion on video calls or in-person meetings, including exchange of knowledge on practical issues encountered during the acquisitions. Moreover, while the images were fully processed locally, the analysts were trained by a central team that had developed the modelling and provided the software. In the initial stages of the project some locally derived results were compared against a central analysis and corrected if needed. As a result, the substudies cannot be considered fully independent, which may have created some bias in the data.

The protocols were set up originally with an aim to match the resolution across all scanners and therefore minimise the risk of differences caused by different levels of partial volume, for instance. However, this resulted in the same low resolution used at 4.7 T being used at 7 T. Another possible limitation is that the sequence parameters in this study were standardised across all field strengths rather than adjusted to account for the field dependency in relaxation times and relaxivity. The flip angle is an exception because one of the centres had inadvertently used an earlier version of the protocol. It is not expected that this would cause significant bias in the results, as all FAs are set to the corresponding value per centre in the signal model used for the analysis. However, the difference may have some effect on the level of B1 effects and possibly inflow suppression. In addition, alternative approaches such as using a slightly bigger field of view rather than phase oversampling were not considered in the initial set up of the sequence. Revisiting the protocol set up in future developments could aid in overcoming some of these limitations.

## Conclusion

Between-centre bias caused by factors such as hardware differences, subject preparations, and operator dependence is the main source of variability in DCE-MRI of liver function in rats, closely followed by biological between-subject differences. Future method development should focus on reducing these sources of error in order to minimise the sample sizes needed to detect more subtle levels of inhibition.

## Supplementary Information

Below is the link to the electronic supplementary material.

Supplementary file1 (PDF 347 KB)

## Data Availability

Data and code presented in this study are openly available in Zenodo, as follows: 1. DCE-MRI datasets and results in CSV format: https://doi.org/10.5281/zenodo.7838397 (published on 17 April 2023). 2. Tracer-kinetic model source code archived from GitHub: https://zenodo.org/record/8372595 (published on 23 September 2023). 3. PMI (Platform for Research in Medical Imaging) v3.1 software: https://doi.org/10.5281/zenodo.4382479 (published on 21 December 2020).
