# Supplementary material for: Assessment of hepatic transporter function in rats using dynamic gadoxetate-enhanced MRI: a reproducibility study
Source: MAGMA. 2024 Aug 6;37(4):697–708. doi: 10.1007/s10334-024-01192-5 (PMC11417070; doi:10.1007/s10334-024-01192-5)
Supplement: Supplementary file 1 — Supplementary file1 (PDF 347 KB) [file 10334_2024_1192_MOESM1_ESM.pdf]

Supplementary Materials

Assessment of hepatic transporter function in rats using dynamic gadoxetate-enhanced MRI: A reproducibility study

Ebony R. Gunwhy[1†*], Catherine D. G. Hines[2†], Claudia Green[3], Iina Laitinen[4,5], Sirisha Tadimalla[6], Paul D. Hockings[4,7], Gunnar Schütz[3], J. Gerry Kenna[8], Steven Sourbron[1‡], John C. Waterton[8,9‡]

[1]*Division of Clinical Medicine, School of Medicine and Population Health, University of Sheffield, United Kingdom*

[2]*GSK, Collegeville, P.A., USA*

[3]*MR & CT Contrast Media Research, Bayer AG, Berlin, Germany*

[4]*Antaros Medical, GoCo House, Mölndal, Sweden*

[5]*Sanofi-Aventis GmbH, Frankfurt, Germany*

[6]*Institute of Medical Physics, University of Sydney, Australia*

[7]*Chalmers University of Technology, Gothenburg, Sweden*

[8]*Bioxydyn Ltd, St. James Tower, Manchester, United Kingdom*

[9]*Centre for Imaging Sciences, Division of Informatics Imaging & Data Sciences, School of Health Sciences, Faculty of Biology Medicine & Health, University of Manchester, United Kingdom*

[†] *These authors contributed equally to this work*

[‡]*These authors jointly supervised this work*

*Corresponding author

1 THE TRISTAN RAT ASSAY: DEFINITION

The TRISTAN rat assay measures the hepatic plasma clearance rate, K^{trans} [mL/min/mL] and biliary efflux rate, k_{bh} [mL/min/mL] of gadoxetate in rats. Guidelines including detailed technical specifications and SOPs on animal handling, data acquisition, and image processing for the TRISTAN rat assay are provided below. Relevant software for image analysis [1] and modelling [2] are also made available.

1.1 SUBJECTS AND PREPARATION

Subjects: male Wistar-Han rats.

Number of subjects: Investigators are permitted to choose the number of rats, balancing the ethical need to minimise animal use while entering sufficient rats to obtain estimates of the uncertainty adequate for the context of use.

Preparation: Animals are allowed to acclimate for 1 week prior to initiation of the imaging study with adequate husbandry. For the scan, animals are anaesthetised with a suitable inhalation anaesthetic such as isoflurane to place a tail vein catheter and maintained under anaesthesia using approximately 2% isoflurane in the air mixture. Animals should be monitored during the scan for respiratory rate and body temperature and provided external heat, e.g., through forced hot air or circulating warm water. Before the start of the exam, animals receive a saline or vehicle control, or a test compound.

1.2 DATA ACQUISITION

MRI is performed on an MRI system suitable for high-quality dynamic rat MRI [3]. Such systems typically operate between 3T and 14T. MRI sequences include localizers, followed by a T_2-weighted (T2W) spin echo sequence for delineation of the anatomy. For DCE, heparinized

saline is placed in the tail vein catheter prior to the bolus of gadoxetate. Gadoxetate is diluted 1:5 in saline and administered at 0.5 mL/kg (25 µmol/kg bodyweight).

Dynamic imaging is performed at approximately 60 sec per time point, and 30 time points are acquired. The first five time points are considered baseline, and the IV bolus of gadoxetate is injected at the beginning of the sixth measurement at 4 min and 45 seconds into the acquisition. The slice selections of the T2W, T_1 map, and dynamic series all use the same slice orientation to facilitate ROI selection.

1.3 IMAGE PROCESSING

MRI images are exported, ideally in DICOM format, and analysed using any suitable software package which permits the operations below. The location of the liver is confirmed on T2W images, and three circular ROIs are placed in three separate slices of liver parenchyma, ventral to the caudate processes and approximately 10 voxels in diameter; the locations of the ROIs are then used to record liver signal intensities averaged over the ROI on the dynamic series. RoIwise rather than voxelwise analysis minimises the risk of biased data at the Rician noise floor. Signal time curves are extracted in csv format and further processed using a python script available here [2]. The script fits the time curves to a model (details below). The three ROI values are then averaged producing a single value for K^{trans} and k_{bh} for each subject at each visit.

1.4 DATA MODELLING

The kinetic model assumes that two separate liver compartments can be distinguished, the extracellular space, e and the intrahepatocytic compartment, h (defined by their respective volumes, v_e and v_h, the mean transit time in h, T_h, and the extraction fraction of gadoxetate from the extracellular to the hepatocyte compartment, E). The mean transit time in the

extracellular space is assumed to be negligible at a sampling time of 58s. With a given arterial input function $c_a(t)$, this produces the following solution for the concentrations in e and h, $c_e(t)$ and $c_h(t)$:

$$c_e(t) = (1 - E)c_a(t) \tag{1}$$

$$v_h c_h(t) = e^{-\frac{t}{T_h}} * E F_p c_a(t) \tag{2}$$

where F_p is the liver plasma flow in mL/min/mL.

Considering the difficulty in measuring an arterial input function reliably in small animals, the TRISTAN rat assay implements a standardised $c_a(t)$ derived from a simplified model of the rat circulation [4] as shown in **Figure 1**. Within this model, literature values for rats are used for all constants as follows: whole body permeability-surface for gadoxetate (PS) was estimated from the rat reproducibility data as 0.172 mL/sec, using the PBPK model from Table 3 in Scotcher et al. [5]; whole body plasma volume (Vp) = (1 – Hct)(V_B) [mL], where Hct is the hematocrit equal to 0.418 [6] and V_B is the whole body blood volume, estimated as 15.8 mL when assuming a body weight of 250 g [7]; whole body extracellular volume (V_E) was derived as 30 mL from Table 2 in the supplementary material in Scotcher et al. [5]; K = GFR + (K^{trans})(V_L), where GFR is the glomerular filtration rate equal to 0.023 mL/sec [8], K^{trans} is the hepatic plasma clearance rate [mL/min/mL], and V_L is the liver volume equal to 8.47 mL [5].

The relaxation rate of liver tissue is derived from the concentrations as follows:

$$R_1(t) = R_{10} + r_{1,p} v_e c_e(t) + r_{1,h} v_h c_h(t) \tag{3}$$

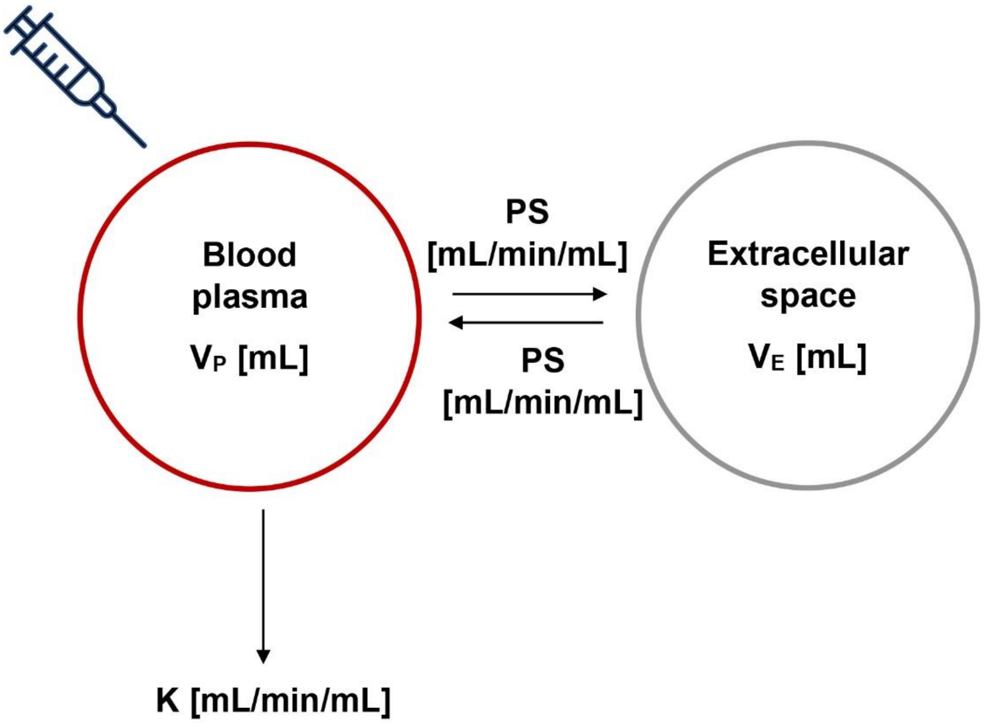

Figure 1. Tracer kinetic model for blood concentrations in rats. PS = whole body permeability-surface for Gadoxetate; V_P = whole body plasma volume; V_E = whole body extracellular volume; K = GFR + (K^{trans})(V_L), where GFR is the glomerular filtration rate, K^{trans} is the hepatic plasma clearance rate, and V_L is the liver volume.

A single consensus pre-contrast longitudinal relaxation rate R_{10} is adopted for each field strength (R_{10} = 1.281 s^{-1} at 4.7T; R_{10} = 1.109 s^{-1} at 7T). This is based on a weighted mean of rat liver R_1 values aggregated with published values [9]. Values for hepatocyte and blood plasma relaxivity ($r_{1,h}$ and $r_{1,p}$) are also obtained from literature [10]: 7.6 s^{-1}/mM and 6.0 s^{-1}/mM for hepatocytes at 4.7T and 7T, respectively, and 6.4 s^{-1}/mM and 6.2 s^{-1}/mM for blood plasma at 4.7T and 7T, respectively. v_e is equally fixed to the literature value of 0.230 [10].

Finally, signals, S are derived from liver relaxation rates assuming the standard signal model of a variable flip angle sequence in steady state:

$$S = \frac{S_0(1 - e^{-TR * R_1(t)})}{1 - \cos(\alpha) e^{-TR * R_1(t)}} \quad (4)$$

where α is the flip angle, TR is the repetition time, S is the signal amplitude in arbitrary units (a.u.) and S_0 (in the same a.u.) is the signal asymptote at infinite TR.

Inserting Eq. (**1**) into Eq. (**2**) and Eq. (**2**) into Eq. (**3**) produces a model for the signal time course S(t). Previous sensitivity analysis [11] has demonstrated that aside from S_0, only two parameters E and T_h can be reliably determined from the given data. Hence, F_p is fixed to a value of 1.32 mL/min/mL, assuming F_p = (1-Hct)*F_b, where F_b is the blood flow = (hepatic artery flow [mL/min] + portal vein blood flow [mL/min]) / mass of the liver [mL], and is calculated as 2.27 mL/min/mL using literature values from Tables 3-4 in the supplementary material of Scotcher et al. [5]. Model fitting for the remaining free parameters S_0, E and T_h is performed by the Python script accessible on Zenodo [2]. The hepatic plasma clearance rate parameter, K^{trans} is derived from K^{trans} = E*F_p and gadoxetate efflux, k_{bh}, is calculated as k_{bh} = v_h/T_h, using 0.722 as the hepatocellular extracellular volume fraction, v_h (calculated from v_h = v_t – v_i, where v_t is the tissue volume fraction = (1 – blood volume fraction) = 0.115 and v_i is the interstitial volume fraction = 0.163 [12]).

1.5 DATA ANALYSIS AND REPORTING

Analysts blinded to treatment perform a quality assessment of the data, considering artefacts or missing data in the scans and goodness-of-fit of the model to the signal time curves. If a single dataset shows strong artefacts or poor goodness-of-fit, the subject should be excluded. For the remaining subjects, K^{trans} and k_{bh} values are averaged to produce a final set of values, and 1.96 x standard error on the mean is returned as the 95% confidence interval (CI). The variance in the standard error must be calculated using Bessel's correction. These numbers are reported to the requesting entity as the rate constants of the drug, with centre-specific uncertainty, as measured by the TRISTAN rat assay. In addition, error intervals must be derived

using the percentage errors measured in this report (reproducibility error). Benchmark values in saline derived in this report should be included as reference.

2 REFERENCES

1. Sourbron S (2020) plaresmedima/PMI-0.4-TRISTAN-RATS: TRISTAN RATS v3.1 (v3.1). Zenodo. doi:10.5281/zenodo.4382479

2. Gunwhy ER, Sourbron S (2023) TRISTAN-rat (v2.0.0). Zenodo. doi:10.5281/zenodo.7839203

3. Waterton JC, Hines CDG, Hockings PD, Laitinen I, Ziemian S, Campbell S, Gottschalk M, Green C, Haase M, Hassemer K, Juretschke HP, Koehler S, Lloyd W, Luo Y, Mahmutovic Persson I, O'Connor JPB, Olsson LE, Pindoria K, Schneider JE, Sourbron S, Steinmann D, Strobel K, Tadimalla S, Teh I, Veltien A, Zhang X, Schutz G (2019) Repeatability and reproducibility of longitudinal relaxation rate in 12 small-animal MRI systems. Magn Reson Imaging 59:121-129.

4. Gunwhy ER, Tadimalla S, Waterton JC, Hockings PD, Schütz G, Green C, Kenna JG, Sourbron S (2023) A novel approach to derive robust arterial input functions for DCE-MRI in small animals. In: Proceedings of the Annual Meeting & Exhibition, International Society of Magnetic Resonance in Medicine, Toronto, ON, Canada. Abstract nr 2761.

5. Scotcher D, Melillo N, Tadimalla S, Darwich AS, Ziemian S, Ogungbenro K, Schütz G, Sourbron S, Galetin A (2021) Physiologically Based Pharmacokinetic Modeling of Transporter-Mediated Hepatic Disposition of Imaging Biomarker Gadoxetate in Rats. Molecular pharmaceutics 18 (8):2997-3009.

6. Cremer JE, Seville MP (1983) Regional brain blood flow, blood volume, and haematocrit values in the adult rat. Journal of cerebral blood flow & metabolism 3 (2):254-256.

7. Lee HB, Blaufox MD (1985) Blood volume in the rat. Journal of Nuclear Medicine 26 (1):72-76.

8. Jobin J, Bonjour J (1985) Measurement of glomerular filtration rate in conscious unrestrained rats with inulin infused by implanted osmotic pumps. American Journal of Physiology-Renal Physiology 248 (5):F734-F738.

9. Waterton JC (2021) Survey of water proton longitudinal relaxation in liver in vivo. Magnetic Resonance Materials in Physics, Biology and Medicine 34 (6):779-789.

10. Ziemian S, Green C, Sourbron S, Jost G, Schutz G, Hines CDG (2021) Ex vivo gadoxetate relaxivities in rat liver tissue and blood at five magnetic field strengths from 1.41 to 7 T. NMR in biomedicine 34 (1):e4401.

11. Tadimalla S, Green C, Steinmann D, Koehler S, Juretschke H-P, Laitinen I, Waterton JC, Hockings PD, Hines CD, Schütz G (2019) Repeatability of hepatocellular uptake and efflux in the rat liver: A comparison of Gadoxetate DCE-MRI models. In: Proceedings of the 27th Annual Meeting & Exhibition, International Society of Magnetic Resonance in Medicine, Montréal, QC, Canada. Abstract nr 5034. https://cds.ismrm.org/protected/19MProceedings/PDFfiles/5034.html.

12. Kawai R, Lemaire M, Steimer J-L, Bruelisauer A, Niederberger W, Rowland M (1994) Physiologically based pharmacokinetic study on a cyclosporin derivative, SDZ IMM 125. Journal of pharmacokinetics and biopharmaceutics 22:327-365.
